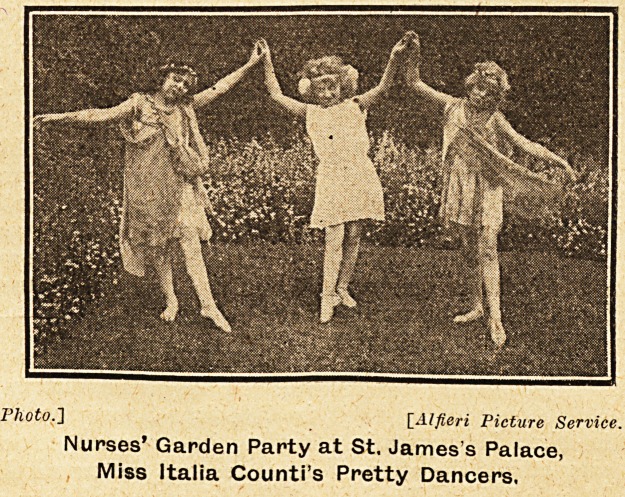# Round the Hospitals

**Published:** 1919-07-05

**Authors:** 


### ROUND THE HOSPITALS.

The *Times* of Wednesday points out that on the day on which war was declared Sir Henry Newbolt wrote on its leader page:

England, on thy knees to-night
Pray that God defend the Right

On Sunday, July 6, we shall rejoice in accepting the summons to record, in solemn thanksgiving, our belief that the Right has prevailed. For nearly five years the worst war of the world has tried us all as a nation and people. Every individual and every interest in the British Islands desires and needs a lengthened period of cessation from strife. From Victory Sunday onwards we hope that peace may prevail throughout the nursing world. The Government, supported by the House of Commons, in dealing with the Central Committee's Bill on

**ROUND THE HOSPITALS—(*continued*).**

Registration (as all can read on pp. 361-8), plainly demonstrated that this Bill could not become an Act of Parliament or pass. The representatives of Lord Goschen's Bill intimated their endorsement of the policy announced by Dr. Addison on behalf of the Government, and the withdrawal of their Registration Bill in the House of Lords. The course is now clear to the Government to set up Nurse Registration upon a basis which will guarantee, impartially, the best interests of all British nurses throughout the country. Every fully trained and intelligent nurse may well welcome the present position, and possess her soul in patience, realising that a sound and acceptable system of Nurse Registration may now be found. So far, well. But the post has brought us evidence that a small section of individuals is seeking opportunities to ape the frog in the fable. They are continuously striving with all their might to create friction and spread insult amongst their sister nurses from whom they may differ in opinion. Let them cease these wanton tactics or the fate of the vain-glorious frog will certainly be theirs. We think it well, therefore, to intimate that THE HOSPITAL will no longer countenance any such foolish virgins. We are assured that all intelligent trained nurses will further our efforts in this direction, for there lies the best interests of the whole profession of nursing. Peace has been signed. Our victory is recognised throughout the nations. Thanksgiving Sunday will be with us when these words are in circulation. And the Right has prevailed. Henceforward let our policy be, *Away with discord and dissension.* We will together seek the sunshine of unity, and warm our hearts with kindness and the co-operation and welcome of our fellow-workers, at home and throughout the world. Those who love discord and Jazz music must seek it elsewhere than in the columns of THE HOSPITAL.

THE debate in the House of Commons on the Registration Bill brought out the painful impression made on the minds of impartial observers by the unhappy tone of acrimonius railing adopted by the supporters of the Central Committee's Bill, its prime mover and organ. We are well aware that this railing is a source of extreme discomfort to many sincere supporters of the Central Committee. They excuse it against their conscience, they ignore it whenever possible, but they neither like nor approve it, directed as it is against many whom they whole-heartedly honour and admire. The cause of State Registration as conceived by the Central Committee has suffered cruelly at the hands of certain of its adherents. Vituperation is not merely an ill in itself, not simply a source of distress to those against whom it is directed, but an ever-open sluice through which the priceless forces of energy and vitality are fruitlessly dissipated. It is sheer waste, worse form, and a vital defect in propaganda, as the action of the House of Commons on June 27 demonstrated.

AT the Investiture held at Buckingham Palace on June 26 the King personally conferred the decoration of the Royal Red Cross on the following members of the nursing services: Bar to the Royal Red Cross: Matron Mary Payne and Matron Margaret Steele, Q.A.I.M.N.S. Royal Red Cross, 1st Class: Matron Florence Spindler, late Nyassaland N.S.; Sister Alberta Armstrong and Sister Amy McMahon, Canadian A.N.S. Royal Red Cross, 2nd Class: Assistant Nurse Helen Henry; Mrs. Lionel Hichens and Assistant Nurse Gwenda Verschoyle Q.A.I.M.N.S.; Sister Agnes McMahon, Q.A.I.M.N.S.R; Sister Mary Drury and Staff Nurse Lily Calvert, T.F.N.S.; Matron Florence Andrews, Matron Annie Burgess, Sister Helen Bennett, and Miss Maud Smith, B.R.C.S.; Sister Annie Baillie; Sister Gertrude Cresswell, Sister Margaret Leamy, Sister Carolyn Little, Sister Myrtle MacMillan, Sister Minnie Misner, Sister Emma Moore, Sister Isobel Muir, Sister Kathleen Panton, Sister Marion Price, Sister Agnes Sutherland, Sister Ada Taylor, and Sister Edith Whitlam, Canadian A.N.S.; Sister Evelyn Hutt and Sister Elma Linklater, Australian A.N.S.; Miss Enid Bevan and Mrs. Jessie Payne-Williams, V.A.D. The King conferred the Military Medal for bravery in the field on Sister Beatrice McNair and Sister Lottie Urquhart, Canadian A.N.S.

AT the Investiture held at Buckingham Palace on June 28 the King personally conferred the decoration of the Royal Red Cross on members of the nursing services as follows:—Royal Red Cross, First Class: Matron Christense Sorenson, Sister Eleanor Jeffries, and Sister Constance Keys, Australian A.N.S. Royal Red Cross, Second Class: Sister Helen Crawford, T.F.N.S.; Matron Grace Morgan and Matron Constance Winterbottom, B.R.C.S.; Sister Alice Kennedy, Canadian A.N.S.; Sister Ellen Bennett-Brown, Sister Jean Brydon, Sister Ethel Dement, Sister Effie Garden, Sister Clarice Green, Sister Nano Nagle, and Sister Valerie Woinarski, Australian A.N.S.; Matron Violet Sharp, East African N.S.; Miss Stella Caulfield, V.A.D.

MISS FRANCES C. SKINNER has been appointed to the post of Registrar of the College of Nursing, vacant by the retirement of Miss Ashdown. Miss Skinner was trained at Addenbrooke's Hospital, Cambridge, and has held the post of ward sister at her training-school, at Selly Oak Infirmary, and at the Royal United Hospital, Bath, and as night superintendent at the West Suffolk General Hospital. Since the war Miss Skinner has had exceptional secretarial experience under the Ministry of National Service, where she was in charge of the register of 40,000 men, and also under the Ministry of Labour, in the Demobilisation of Officers' Department. Miss Skinner's familiarity with the card-index system and registration work generally qualify her exceptionally for the responsible position of Registrar of the College of Nursing. She is at present in charge of the College Appointments Bureau. We are advised that the post of assistant secretary will not be filled until after the holidays.

**ROUND THE HOSPITALS—(*continued*).**

Sir R. Douglas Powell appeals in *The Times* for a service of visiting nurses for middle-class patients. He calls attention to the increasing need among the general middle-class public for this class of nursing in chronic and other cases where a visit once or perhaps twice a day from a nurse would enable relatives to carry on in accordance with doctor's orders. We believe that Dr. Powell is mistaken in imagining that V.A.D. help could be utilised for this purpose. It requires a skilled woman to direct, and this must be the function of the visiting nurse. Moreover, the visiting nurse ought not in our judgment to be a kind of free lance, working on her own account with her own clientele. People have an inconsiderate way of all requiring the nurse at the same moment, and the solitary visiting nurse has either nothing to do or is compelled to reject half her calls. A good organising centre is needed for the supply of visiting nurses in all those neighbourhoods where their services are required.

The Hon. Organising Secretary of the Edith Cavell Homes of Rest writes to inform us that "none of the nurses received into our homes pay the cost of their maintenance, as our homes are entirely free, with this exception—some nurses receive from their hospitals a special billeting allowance while on their leave, and we were advised by the principal matrons to suggest that such nurses should contribute the sum of 10s. a week. This is quite optional, and as a matter of fact few nurses do so." We regret the error, which arose from a reference in the published statement of accounts to nurses' payments, doubtless of the character alluded to above. The Honorary Secretary also informs us that the question of founding a sanatorium for phthisical nurses under the Edith Cavell Homes of Rest Committee has been two or three times discussed. It has, however, been decided that as the fund is raised only to provide rest for nurses, and not medical treatment, the provision of a sanatorium does not come under their objects, and the money subscribed to the fund is not subscribed with that intention.

The need for a special sanatorium for nurses is indisputable, and we trust it may attract the attention of the managers of the Nation's Tribute Fund. It could be to some extent self-supporting, since Army nurses are entitled to payment for their treatment by the War Office. It is possible that a bed or beds might be supported by the Junius S. Morgan Fund for the use of members of the Royal National Pension Fund for Nurses who need this costly form of treatment. A well-elaborated scheme, formed in the interests of the nursing profession, would require a considerable measure of voluntary effort to set going outdoor industries and interests and open up new careers for nurses attacked by phthisis in the early stages. Existing sanatoriums for women are either too expensive to be resorted to by nurses or are unsuited to them for other reasons. We trust it may not be very long before they have one of their own in every way worthy of their profession.

Urgent appeals are being made for the Russians in their present extremity of misery, and Princess Christian, as Chairman of the British Committee of the Russian Red Cross in Great Britain, asks for helpers of every kind. Surgeons, doctors, and nurses willing to serve in Russia under this committee are urgently needed. The latter are asked to apply, between 11 and 1, to Lady Egerton, 35 Albemarle Street, W. 1, where full particulars will be given.

Photo.] [Alfieri Picture Service.

**Viscountess Stopford's "Cries of London."**

An interesting visit has recently been paid to the Army on the Rhine by the two Matrons-in-Chief of the Army, Dame E. H. Becher, G.B.E., and Dame E. M. McCarthy, G.B.E. According to the *British Medical Journal* these matrons have the proud distinction of being the only two departmental chiefs who have remained in the same office during the whole war. Dame Becher was responsible for the supply of nurses at home and abroad, including every expedition from German East Africa to Murmansk, while Dame McCarthy performed the same office in France and Flanders. They have met with a hearty welcome on the Rhine.

Less than fifty nurses took part in the women's rally in aid of the Victory Loan, which took place in Trafalgar Square on June 28, and passed irresistibly into a great demonstration of joy as the news reached the crowd that the fateful signatures of the delegates had been appended to the Treaty.

**ROUND THE HOSPITALS—(*continued*).**

Much can be done, both by matrons in institutions and by private nurses among each other, to make known the easy terms on which Victory and Funding Loan can be obtained. Few things are more distressing in the general unbusinesslike habits of nurses than their penchant for what are called "wild cat" investments. For the hope of an extra pound or two a year they will entrust their precious savings to any enterprise which touches their imagination, often to find after a few high dividends have been paid that the capital is lost. Any nurse who buys or induces others to buy the Victory Loan is doing a double service: she secures a first-rate investment for hard-earned money which might otherwise go astray, and she helps her country as surely as when she braved the enemy's bombs.

AMONG the hospital experiments which the War occasioned, and which met with a measure of success, was that of the part-time nurse. An instance of this was the hospital staffed by girls from the mills in North-East Lancashire, who spent one week out of every four in hospital, and for the remaining three returned to the mill. This led to a system by which those at the mill agreed to pool their wages, and divided the total with those at work in the hospital. As an emergency plan it deserves to be recorded, and shows what can be contrived in case of need. We all remember that the eight-hours' day for nurses now coming into vogue was once declared to be impracticable, but that seems a trifle to the enrolment of a fresh section of mill girls every week.

THE directors of the Royal Hospital for Sick Children, Glasgow, announce in the annual report that they have instituted a new and greatly improved curriculum for probationer nurses, "which should be of the greatest advantage to them when they leave the hospital as trained nurses." It would have been better, perhaps, to have specified that the course is directed, in accordance with the objects of the institution, to "the training of nurses *for sick children*." The high standard maintained in this hospital renders the nurses particularly valuable assistants in the infantile mortality and child welfare schemes set on foot in the city of Glasgow and the neighbouring counties, and we learn with satisfaction that the co-operation of the directors has been invited by the local authorities in the direction not only of treatment, but of research work and preventive measures.

AT the Brighton Poor-Law Infirmary a temporary staff nurse has been appointed from Denmark Hill and staff nurses from Twickenham and Croydon, thus bringing into prominence the fact that the probationer nurses at the infirmary do not receive sufficient training to fit them for promotion. It was stated in excuse that, owing to the War Office demands, there was no accommodation for the proper training of nurses. This excuse will not serve much longer, and we trust the Guardians will at once begin to put their house in order. The temporary staff nurse was appointed at £27 a year, but there is a war bonus, which increases the salary to £46 10s. As this is the minimum likely to attract any staff nurse worth having, the Guardians would be well advised to drop this clumsy way of pretending that salaries have not gone up permanently.

THE custom of giving a ration allowance to some members of the nursing service during their holidays is on the increase in institutions, the Portsmouth Guardians being the latest to make this welcome grant. The high cost of food has made holidays an expensive luxury in the case of the resident staff, and many a poor nurse without relatives to invite her home is placed in a very awkward position during her annual leave. The boon of a ration allowance makes all the difference as to the reality of the rest in such cases. Even the bestowal of one day in seven on the nurse has its drawbacks if she feels obliged to spend her day away, and has to provide all her meals. In the Metropolitan Asylum Board fever hospitals, where it is plainly desirable for nurses to spend their days off duty away from the premises if possible, the staff are permitted to draw a money ration in lieu of meals. Some such arrangement in voluntary institutions would be a boon to many.

THE nurses at the St. Albans Infirmary are making just complaints of the cubicles in which they are housed, and the infirmary staff committee are faced with the need for constructing proper nurses' quarters in the near future. They have been warned by one of their own members that the nurses will put up with things as they are for a time, but not for a permanency, and that unless there is reconstruction no fresh probationers will soon be obtainable. A lady guardian pleaded sensibly for the nurses to have a little privacy secured to them after all their hard work, and the Chairman gave a sympathetic though indefinite reply. The subject ought to be tackled in a business-like way forthwith.

*Photo.*] [*Alfieri Picture Service.*

**Nurses' Garden Party at St. James's Palace, Miss Italia Counti's Pretty Dancers.**

## Figures and Tables

**Figure f1:**
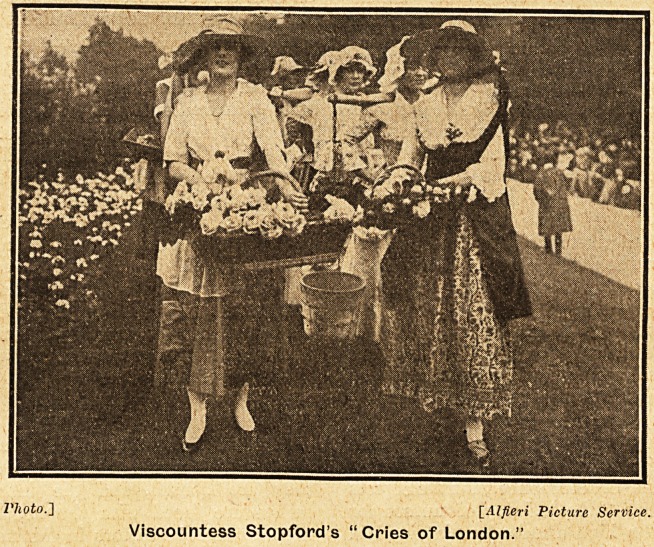


**Figure f2:**